# A Case–Control Seroprevalence Study on the Association Between *Toxoplasma gondii* Infection and Bipolar Disorder

**DOI:** 10.3389/fpsyt.2019.00766

**Published:** 2019-10-25

**Authors:** Cosme Alvarado-Esquivel, Sergio Estrada-Martínez, Alma Rosa Pérez-Alamos

**Affiliations:** ^1^Faculty of Medicine and Nutrition, Juárez University of Durango State, Durango, Mexico; ^2^Institute for Scientific Research “Dr. Roberto Rivera Damm,” Juárez University of Durango State, Durango, Mexico

**Keywords:** *Toxoplasma gondii*, seroprevalence, bipolar disorder, psychiatric patients, case–control study

## Abstract

**Background and Aims:** Infection with the parasite *Toxoplasma gondii* has been associated with bipolar disorder in several countries other than Mexico. Therefore, we sought to determine the association between seropositivity to *T. gondii* and bipolar disorder in a Mexican population.

**Methods:** We performed an age- and gender-matched case–control study of 66 patients with bipolar disorder (WHO *International Classification of Diseases, 10th Revision* code: F31) and 396 subjects without this disorder from the general population. Anti-*Toxoplasma* immunoglobulin G (IgG) and IgM antibodies were determined using commercially available enzyme-linked immunoassays.

**Results:** Six (9.1%) of the 66 patients with bipolar disorder and 22 (5.6%) of the 396 controls had anti–*T. gondii* IgG antibodies (odds ratio [OR] = 1.7; 95% confidence interval [CI] = 0.66–4.36; *P* = 0.26). Stratification by gender and age did not show a difference in seroprevalence between cases and controls. The frequency of high (> 150 international units/ml) anti–*T. gondii* IgG levels was similar in cases (n = 2) and in controls (n = 12) (OR = 1.0; 95% CI = 0.21–4.57; *P* = 1.00). Stratification by *International Classification of Diseases, 10th Revision* F31 codes showed that patients with F31.3 code had a higher seroprevalence of *T. gondii* infection than their age- and gender-matched controls (OR = 16.4; 95% CI = 1.25–215.09; *P* = 0.04). None of the six anti–*T. gondii* IgG–seropositive patients with bipolar disorder and 4 (18.2%) of the 22 anti–*T. gondii* IgG–seropositive controls had anti–*T. gondii* IgM antibodies (*P* = 0.54).

**Conclusions:** Our results suggest that *T. gondii* seropositivity is not associated with bipolar disorder in general. However, a specific type of bipolar disorder (F31.3) might be associated with *T. gondii* seropositivity. Further research to elucidate the role of *T. gondii* infection in bipolar disorder is needed.

## Introduction

*Toxoplasma gondii* is one of the most successful intracellular parasites with strategies to avoid destruction by the host and to obtain lifelong survival (1). This pathogen that infects over one-third of the global human population invades and chronically persists in the central nervous system of the infected host (2). Most human infections are mild or asymptomatic; however, *T. gondii* infection can result in life-threatening disease in immunocompromised individuals (3). Primary infection with *T. gondii* in pregnant women may cause abortions and central nervous and eye disease in the fetus leading to disability (4). Chronic infection in the brain correlates with changes in neuronal architecture, neurochemistry, and behavior suggesting that chronic infection is not without consequence (5). Prevalence of infection with *T. gondii* has been found higher in psychiatric patients than in controls (6–8). Infections with *T. gondii* have been associated with suicide attempts (9–11), mixed anxiety and depressive disorder (12), schizophrenia (13–15), depression (16), and obsessive–compulsive disorder (13). There is increasing evidence of an association between infection with *T. gondii* and bipolar disorder. Studies in several countries have found a higher seroprevalence of *T. gondii* infection in patients suffering from bipolar disorder than in controls (17–22). In contrast, no association between maternal infection with *T. gondii* and risk of bipolar disorder in offspring was found (23, 24). Bipolar disorder is a public health problem around the worldwide, and about 1% of the population suffers from this disease (25, 26). To the best of our knowledge, the association between *T. gondii* infection and bipolar disorder has not been studied in Mexican populations. Therefore, we sought to determine the association between seropositivity to *T. gondii* infection and bipolar disorder in Durango City, Mexico.

## Materials and Methods

### Study Design and Population

Through an age- and gender-matched case–control study design, we studied 66 psychiatric patients suffering from bipolar disorder attended in a public hospital of mental health (Hospital of Mental Health “Dr. Miguel Vallebueno” of the Secretary of Health) in the northern Mexican city of Durango and 396 control subjects without bipolar disorder from the general population of the same city. Inclusion criteria for enrollment of cases were (1) patients suffering from bipolar disorder diagnosed in the Hospital of Mental Health “Dr. Miguel Vallebueno”; (2) 18 years or older; and (3) who voluntarily accepted to participate in the survey. Bipolar disorder was diagnosed by psychiatrists and was classified according to the classification of mental and behavioral disorders of the *International Classification of Diseases, 10th Revision* (*ICD-10*) (https://www.icd10data.com/ICD10CM/Codes/F01-F99/F30-F39/F31-). Bipolar disorder has the *ICD-10* code F31. Blood sampling of subjects was performed at the time that corresponds to the most recent diagnostic *ICD-10* code F31. Of the 66 patients with bipolar disorder, 33 (50.0%) were females and 33 (50.0%) were males. Their mean age was 40.05 ± 14.48 (range, 20–76) years. Control subjects were obtained from the general population of Durango City, selected at random, and matched with cases for gender and age (± 2 years). Inclusion criteria for enrollment of controls were (1) subjects of the general population of Durango City without bipolar disorder; (2) 18 years or older; and (3) who voluntarily accepted to participate in the survey. Of the 396 controls, 198 (50.0%) were females and 198 (50.0%) were males. Mean age in controls subjects was 40.05 ± 14.39 (range, 20–77) years. Age and gender in cases were similar to those in controls (*P* = 0.99, and *P* = 1.0, respectively). The socioeconomic status of participants was not included in the matching of cases and controls because this characteristic has not been impacting on the seroprevalence of *T. gondii* infection in the region.

### Laboratory Tests for Detection of Anti–*T. Gondii* Immunoglobulin G and Immunoglobulin M Antibodies

Each participant provided a blood sample. After centrifugation of blood samples, serum samples were obtained and kept frozen at −20°C until analyzed. Serum samples of participants were analyzed for detection of anti–*T. gondii* immunoglobulin G (IgG) antibodies with the commercially available enzyme immunoassay (EIA) kit “*Toxoplasma* IgG” (Diagnostic Automation/Cortez Diagnostics Inc., Woodland Hills, CA, USA). This assay has a cutoff of ≥8 international units (IU)/ml of anti–*T. gondii* IgG antibody. Serum samples of participants who were seropositive for anti–*T. gondii* IgG antibodies were further examined for detection of anti–*T. gondii* IgM antibodies by the commercially available EIA “*Toxoplasma* IgM” kit (Diagnostic Automation/Cortez Diagnostics Inc.). All IgG and IgM assays were performed according to the instructions of the manufacturer.

### Statistical Analysis

Statistical analysis of the data was performed with the aid of the software Epi Info version 7 and the SPSS version 15.0 (SPSS Inc., Chicago, IL). We calculated the sample size using the following values: a reference seroprevalence of *T. gondii* infection of 6.1% (27) as the expected frequency of exposure in controls, a power of 80%, a confidence level of 95%, a 1:6 proportion of cases and controls, and an odds ratio (OR) of 3.5. We used this high OR because some population groups in Durango City (12, 15) have about threefold or fourfold higher seroprevalences of *T. gondii* infection than the 6.1% seroprevalence found in the general population in the same city (27). However, using these values, an association can be detected even below a 3.5 OR. The result of the sample size calculation was 54 cases and 320 controls. The Student *t* test was used to compare the ages among cases and controls. We used the two-tailed Pearson χ^2^ test or the Fisher exact test (when values were small) to determine the association of *T. gondii* infection and bipolar disorder. In addition, we calculated the OR and 95% confidence interval (CI), and statistical significance was set at *P* < 0.05.

### Ethical Aspects

This project was approved by the Ethics Committee of the General Hospital of the Secretary of Health in Durango City, Mexico. Subjects were invited to voluntarily participate in the study, and they were informed about the purpose and procedures of this survey. A written informed consent was obtained from each participant.

## Results

Six (9.1%) of the 66 patients with bipolar disorder and 22 (5.6%) of the 396 controls had anti–*T. gondii* IgG antibodies. The difference in seroprevalence of *T. gondii* infection between cases and controls was not statistically significant (OR = 1.7; 95% CI = 0.66–4.36; *P* = 0.26). Further analysis with stratification by sex and age did not show a difference in seroprevalence between cases and controls (**Table 1**). Of the six anti–*T. gondii* IgG–positive cases, two (33.3%) had IgG levels higher than 150 IU/ml, two (33.3%) between 100 and 150 IU/ml, and two (33.3%) between 8 and 99 IU/ml. On the other hand, of the 22 anti–*T. gondii* IgG–positive controls, 12 (54.5%) had IgG levels higher than 150 IU/ml, two (9.1%) between 100 and 150 IU/ml, and 8 (36.4%) between 8 and 99 IU/ml. The frequency of high (> 150 IU/ml) anti–*T. gondii* IgG levels was similar in cases and controls (OR = 1.0; 95% CI = 0.21–4.57; *P* = 1.00).

**Table 1:** Stratification by sex and age in cases and controls and seropositivity rates to *Toxoplasma gondii*.

Characteristics	Cases			Controls			Odds ratio	95% CI	*P* value
	Seropositivity to *T. gondii*			Seropositivity to *T. gondii*					
	No. tested	No.	%	No. tested	No.	%			
Sex									
Male	33	3	9.1	198	10	5.1	1.88	0.48–7.22	0.35
Female	33	3	9.1	198	12	6.1	1.55	0.41–5.81	0.51
Age (years old)									
≤30	22	1	4.5	132	2	1.5	3.00	0.26–35.6	0.34
31–50	24	3	12.5	144	13	9.0	1.44	0.37–5.48	0.59
> 50	20	2	10.0	120	7	5.8	1.79	0.34–9.32	0.48

Stratification by *ICD-10* F31 diagnosis groups showed that patients suffering from bipolar disorder, with current episode depressed, of mild or moderate severity (F31.3) had a higher seroprevalence of anti–*T. gondii* IgG antibodies than their age- and gender-matched controls (OR = 16.4; 95% CI = 1.25–215.09; *P* = 0.04) (**Table 2**). All but one of the patients had only one psychiatric illness (bipolar disorder). One patient had two psychiatric illnesses: bipolar disorder and schizoaffective disorder.

**Table 2:** Comparison of *Toxoplasma gondii* seropositivity rates in cases and controls according to *International Classification of Diseases, 10th Revision* (*ICD-10*) diagnosis groups.

ICD-10 groups	Diagnosis	Cases				Controls		
		Prevalence of *T. gondii* infection				Prevalence of *T. gondii* infection		
		No.	No.	%	No.	No.	%	*P*
F31.0	Bipolar disorder, current episode hypomanic	35	4	11.4	210	15	7.1	0.49
F31.1	Bipolar disorder, current episode manic without psychotic features	12	0	0.0	72	3	4.2	1
F31.1 + 25.1	Bipolar disorder, current episode manic without psychotic features + schizoaffective, depressive type	1	0	0.0	6	2	33.3	1.00
F31.2	Bipolar disorder, current episode manic severe with psychotic features	6	0	0.0	36	0	0.0	—
F31.3	Bipolar disorder, current episode depressed, mild or moderate severity	7	2	28.6	42	1	2.4	0.04
F31.4	Bipolar disorder, current episode depressed, severe, without psychotic features	3	0	0.0	18	1	5.6	1.00
F31.5	Bipolar disorder, current episode depressed, severe, with psychotic features	1	0	0.0	6	0	0.0	—
F31.9	Bipolar disorder, unspecified	1	0	0.0	6	0	0.0	—

Concerning anti–*T. gondii* IgM seropositivity, none of the six anti–*T. gondii* IgG–seropositive patients with bipolar disorder had anti–*T. gondii* IgM antibodies. In contrast, anti–*T. gondii* IgM antibodies were found in 4 (18.2%) of the 22 anti–*T. gondii* IgG–seropositive controls. No statistically significant difference in anti–*T. gondii* IgM seropositivity rates in cases and in controls was found (*P* = 0.54).

## Discussion

After infection, *T. gondii* disseminates to a large variety of organs in the body including brain (28). Studies in several countries other than Mexico have reported an association between seropositivity to *T. gondii* and bipolar disorder. However, we are not aware of any study about this association in Mexico. Therefore, we performed this case-control study to determine whether seropositivity to *T. gondii* is associated with bipolar disorder in a group of patients in the northern Mexican city of Durango. We found that patients suffering from bipolar disorder had a similar seroprevalence of *T. gondii* infection to age- and gender-matched control subjects. We did not find a difference in IgG or IgM seropositivity rates among cases and controls. In addition, the frequency of high levels of anti–*T. gondii* IgG antibodies was similar in cases and controls. Therefore, our results based on IgG and IgM seroprevalence and IgG serointensity suggest that *T. gondii* infection is not associated with bipolar disorder in patients in Durango, Mexico, in general. The seroprevalence (9.1%) found in patients with bipolar disorders is comparable with the seroprevalences of *T. gondii* infection reported in general population (6.1%) (27) and blood donors (7.1%) in the same Durango City (29). Furthermore, the seroprevalence found in patients suffering from bipolar disorder is lower than seroprevalences reported in high-risk population groups including waste pickers (21.1%) (30), schizophrenic patients (20%) (15), and inmates (21.1%) (31) also in the same Durango City. This comparison further supports the lack of association of *T. gondii* infection and bipolar disorder in our setting. However, we found an association between *T. gondii* seropositivity and patients suffering from bipolar disorder, with current episode depressed, of mild or moderate severity (F31.3). This association should be interpreted with caution since the 95% CI obtained was very wide. The sample size of patients with this code was very small, and further studies with large sample sizes of these patients to elucidate the association should be conducted. It is not clear why *T. gondii* infection was not associated with bipolar disorder in this study in general. In fact, the lack of association between *T. gondii* infection and bipolar disorder found in the current study was unexpected. Other studies have reported a higher seroprevalence of *T. gondii* infection in patients suffering from bipolar disorder than in control subjects. These findings have been reported in Saudi Arabia (19), France (20, 32), the United States (22), and Ethiopia (17). In a meta-analysis of 50 studies about the association of seroprevalence of *T. gondii* infection and psychiatric disorders, researchers found a significant OR with IgG antibodies in bipolar disorder (13). We are not aware of a previous age-and gender-matched case–control study that reported a lack of association between *T. gondii* infection and bipolar disorder. Differences in the association between *T. gondii* infection and bipolar disorder among the studies could be explained by differences in the characteristics of the patients studied including severity and duration of the disease, presence of concomitant psychiatric disease as schizophrenia, or duration of the infection (acute or chronic). All *T. gondii*–seropositive patients in our study had latent infection, and it is not clear whether clinical features of bipolar disorder might be associated only with acute *T. gondii* infection or chronic infection or both. We studied only patients attended in a public hospital, whereas other researchers have studied a population-based sample of subjects (22).

The present study has some limitations: the study population of patients with bipolar disorder was enrolled in only a psychiatric hospital, we studied only adult patients, and the sample size of specific *ICD-10* F31 diagnosis groups was small. Additional studies to assess the association between *T. gondii* seropositivity and bipolar disorder should enroll not only adults but also younger patients in several hospitals and include a large sample size of patients with *ICD-10* F31 diagnosis codes. Our study was performed in a low *T. gondii* seroprevalence population, and this condition may help to identify only clear associations when using small sample sizes, as we found in previous studies of 50 schizophrenic patients (15) and 65 patients with mixed anxiety and depressive disorder (12). However, it is possible that using a larger sample size may help to identify an association between *T. gondii* infection and bipolar disorder in our region.

## Conclusions

Our results suggest that *T. gondii* seropositivity is not associated with bipolar disorder in general. This finding conflicts with those reported in previous studies. However, a specific type of bipolar disorder (F31.3) might be associated with *T. gondii* seropositivity. Further research to elucidate the role of *T. gondii* infection in bipolar disorder is needed. Data of the present study can be useful for further research to determine the association between *T. gondii* infection and bipolar disorder using a meta-analysis design.

## Data Availability Statement

The raw data supporting the conclusions of this article will be made available by the authors, without undue reservation, to any qualified researcher.

## Ethics Statement

The studies involving human participants were reviewed and approved by Ethics Committee of the General Hospital of the Secretary of Health in Durango City, Mexico. The patients/participants provided their written informed consent to participate in this study.

## Author Contributions

CA-E conceived and designed the study, obtained blood samples, performed laboratory tests and data analysis, and wrote the manuscript. SE-M performed the statistical analysis and reviewed the manuscript. AP-A obtained blood samples, summited questionnaires, and reviewed the manuscript.

## Funding

This study was financially supported by the Secretary of Public Education, Mexico (grant no. DSA/103.5/14/11311).

## Conflict of Interest

The authors declare that the research was conducted in the absence of any commercial or financial relationships that could be construed as a potential conflict of interest.
